# Coexistence of a Hemispheric Developmental Venous Anomaly and Two Ipsilateral Arteriovenous Malformations: A Rare Neurovascular Association

**DOI:** 10.7759/cureus.114524

**Published:** 2026-08-14

**Authors:** Karima Sif Nasr, Elmahdi Ait Belhaj, Amine Cherraqi, Amina Elkhamlichi, Meriem Fikri

**Affiliations:** 1 Radiology, Mohammed V Military Hospital, Rabat, MAR; 2 Radiology, Centre Hospitalier Universitaire (CHU) Ibn Sina Hospital, Rabat, MAR; 3 Neuroradiology, Hospital of Specialties, Rabat, MAR

**Keywords:** angiography, dva, hemorrhage, mav, mri

## Abstract

Although developmental venous anomalies (DVAs) are commonly associated with cavernous malformations, their coexistence with arteriovenous malformations (AVMs) remains rare. The presence of multiple AVMs associated with a large hemispheric DVA is exceptional and raises important diagnostic and therapeutic considerations. We present a rare case of this association and discuss its imaging features and clinical implications in light of the existing literature.

A 62-year-old patient presented with right upper limb tremor and memory disturbances. Magnetic resonance angiography revealed a right hemispheric DVA with suspicion of an associated frontal AVM. Digital subtraction angiography confirmed a large right hemispheric DVA associated with two ipsilateral AVMs: a right frontal AVM (Spetzler-Martin grade I) and a right paracentral AVM (grade III), harboring a small intranidal arterial aneurysm. An additional saccular aneurysm arising from the right pericallosal artery was also identified. The patient declined treatment and was managed conservatively. During follow-up, intermittent headaches occurred; emergency CT angiography showed no hemorrhage and stable findings, supporting continuation of the surveillance strategy.

This rare association highlights the complexity of mixed cerebrovascular malformations. Multimodal imaging, particularly digital subtraction angiography (DSA), is essential for accurate diagnosis and risk assessment. Management should be individualized, with strict preservation of DVA venous drainage to avoid severe complications.

## Introduction

Cerebrovascular malformations encompass a heterogeneous group of vascular anomalies of the brain, ranging from benign incidental findings to life-threatening lesions requiring urgent intervention. Among these, arteriovenous malformations (AVMs) and developmental venous anomalies (DVAs) are two distinct entities with different pathophysiological profiles and clinical implications.

AVMs are high-flow intracranial vascular lesions composed of a tangle of abnormal blood vessels--feeding arteries, a central nidus, and draining veins--forming direct arteriovenous shunts without an intervening capillary bed. In practical terms, this abnormal short-circuit exposes thin-walled venous structures to high arterial pressure, predisposing to rupture and intracranial hemorrhage. Clinically, AVMs may present with hemorrhage, seizures, headaches, or focal neurological deficits, and hemorrhagic presentation is associated with substantial morbidity and mortality [1].

DVAs, in contrast, are not true malformations but rather an extreme anatomical variant of normal cerebral venous drainage. They consist of dilated medullary veins converging centripetally into a large collecting vein, producing the classic "caput medusae" appearance on imaging, and they drain the normal surrounding brain parenchyma. DVAs are the most common intracranial vascular malformation, with an estimated prevalence of 2-4% in clinical cohorts. Unlike AVMs, isolated DVAs are associated with a very low annual hemorrhage risk of approximately 0.22% per year and are most often discovered incidentally on brain imaging [2,3].

The coexistence of DVAs and AVMs is rare and remains poorly understood. Their clinical importance arises primarily from hemodynamic interactions: the high-flow arteriovenous shunting generated by AVMs increases venous pressure within the DVA drainage system, which, given the thin-walled and low-pressure nature of DVA veins, may predispose to venous hypertension, thrombosis, or rupture. This interaction renders the DVA no longer an incidental finding but a functionally critical component of the vascular network, with direct implications for clinical behavior and management. We present a rare case of a large hemispheric DVA associated with two ipsilateral AVMs, highlighting the role of multimodal imaging in the diagnosis and management of these complex vascular malformations [1-3].

## Case presentation

We report the case of a 62-year-old man with no significant past medical history who was referred to the neurology department for evaluation of right upper limb tremor associated with memory disturbances. Neurological examination was unremarkable except for tremor involving the right upper extremity. Laboratory investigations were within normal limits.

**Figure 1:**
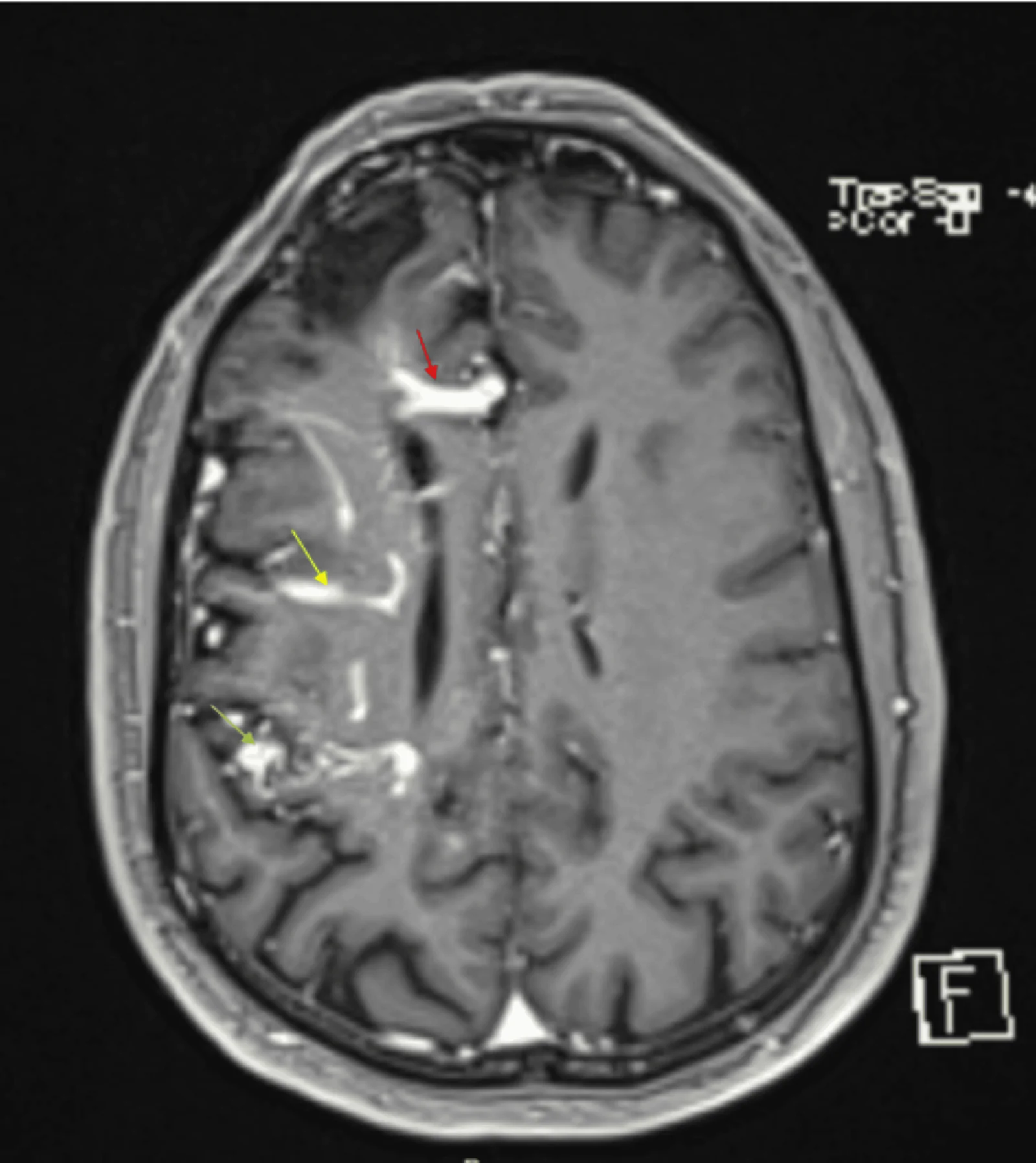
Brain MRI Post-contrast axial T1-weighted image demonstrating multiple, radially oriented medullary veins (yellow arrow) converging into a dilated draining vein (red arrow), consistent with a right hemispheric developmental venous anomaly (DVA). Note the AVM (green arrow). AVM: arteriovenous malformation

**Figure 2:**
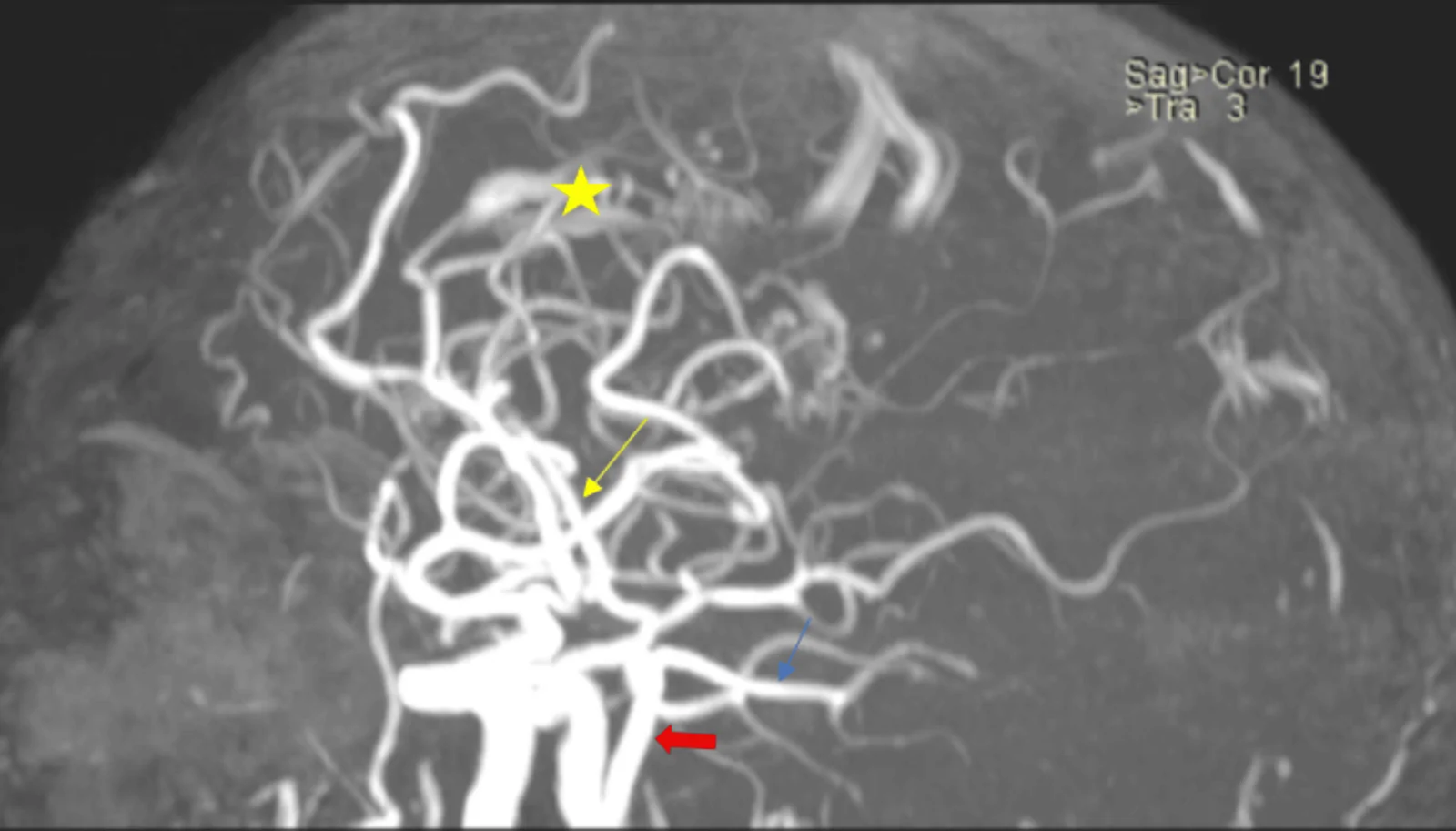
Time-of-flight MR angiography showing abnormal vascular flow-related signal voids suggestive of associated AVMs (star) Note the ACA (yellow arrow), the MCA (blue arrow), and the left internal carotid ( red arrow) AVM: arteriovenous malformation, ACA: anterior cerebral artery; MCA: middle cerebral artery

Magnetic resonance angiography (MRA) demonstrated a large developmental venous anomaly (DVA) involving the right cerebral hemisphere. The lesion was characterized by multiple radially oriented medullary veins converging into a markedly dilated frontal cortical collector vein, producing a typical “caput medusae” appearance. No early venous drainage suggestive of an arteriovenous shunt was identified within the DVA itself. However, an associated ipsilateral frontal arteriovenous malformation (AVM) was suspected. No imaging evidence of intracranial hemorrhage was observed (Figures 1-2).

**Figure 3:**
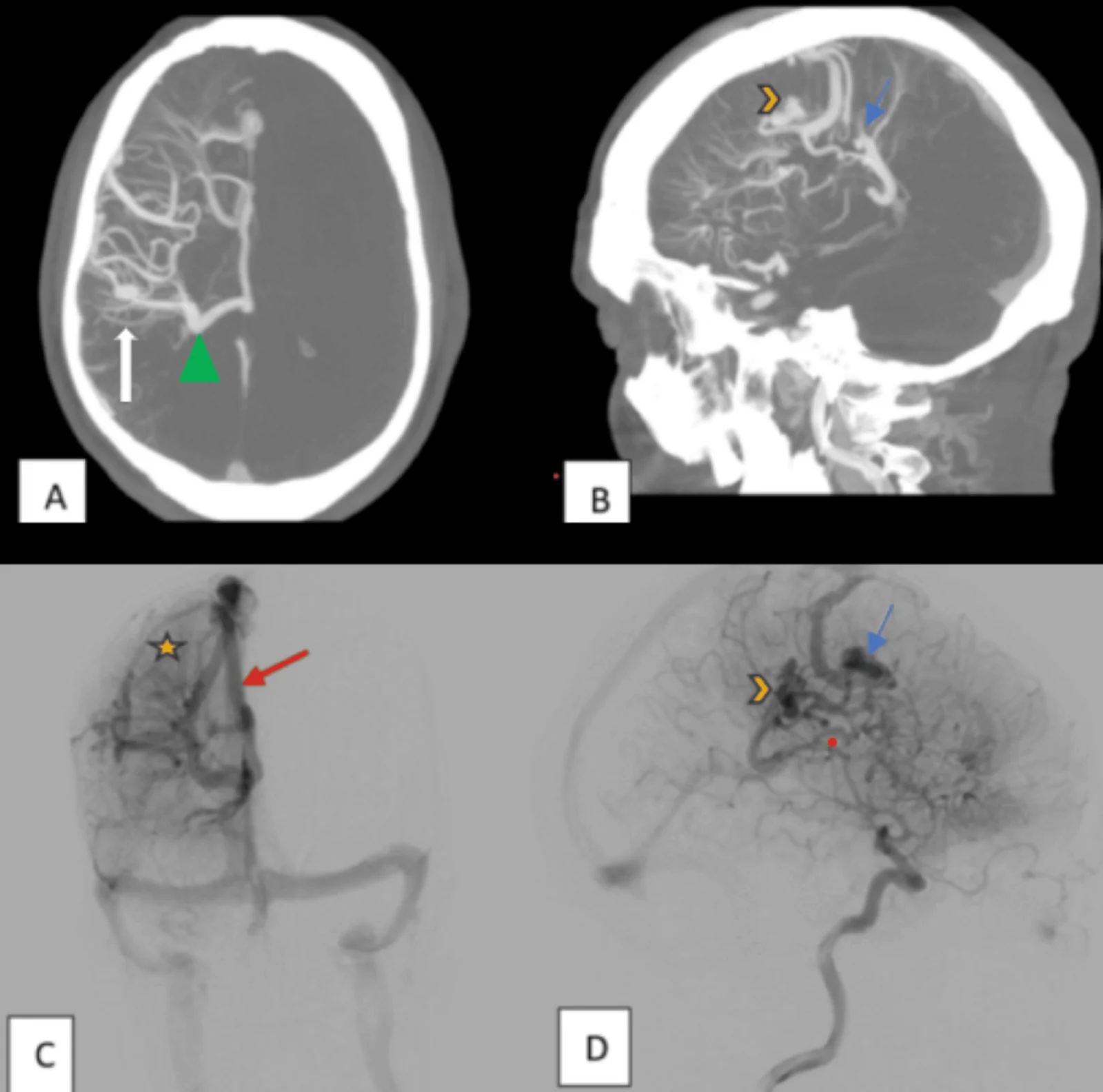
3D reconstructed CT angiograms (A, B) demonstrating two right-sided AVMs located in the frontal (head arrow) and paracentral (blue arrow) regions In image A, the afferent arteries (white arrow) and the draining vein (green arrowhead) of the paracentral AVM are depicted. Cerebral angiography in anteroposterior (C) and oblique (D) projections shows multiple hemispheric radial veins converging into a dilated cortical collector vein and draining into the superior sagittal sinus (red arrow), creating the characteristic 'caput medusae' appearance (star) of a DVA. AVM: arteriovenous malformation; DVA: developmental venous anomaly

Given the suspicion of an associated arteriovenous shunt and for detailed angioarchitectural assessment, diagnostic digital subtraction angiography (DSA) was performed (Figure 3). DSA demonstrated two right supratentorial AVMs.

The first AVM was identified in the right frontal region and consisted of a compact nidus measuring 14 mm. Arterial feeders were derived from branches of the precentral artery arising from the superior division of the ipsilateral middle cerebral artery (MCA). Venous outflow was directed through a moderately dilated collector vein into a dilated cortical vein, draining into the superior sagittal sinus. This lesion was classified as Spetzler-Martin grade I.

The Spetzler-Martin grading system assigns points based on three criteria: nidus size (small <3 cm = 1 point, medium 3-6 cm = 2 points, large >6 cm = 3 points), eloquence of adjacent brain (non-eloquent = 0, eloquent = 1 point), and pattern of venous drainage (superficial only = 0, deep = 1 point). For this lesion, one point was assigned for nidus size (<3 cm), zero points for location in a non-eloquent frontal region, and zero points for exclusively superficial venous drainage, yielding a Spetzler-Martin grade I.

The second AVM was identified in the right paracentral region and exhibited a compact nidus measuring 17 mm in its largest diameter. Arterial supply originated from branches of the central artery arising from the superior division of the ipsilateral MCA. Venous drainage was provided by moderately dilated collector veins, draining both superficially into the previously described cortical vein toward the superior sagittal sinus and deeply into the right internal cerebral vein, subsequently reaching the vein of Galen and the straight sinus. This AVM was associated with a small prenidal arterial aneurysm measuring 1.92 × 1.87 mm and was classified as Spetzler-Martin grade III. One point was assigned for nidus size (<3 cm), one point for eloquent location adjacent to the primary sensorimotor cortex, and one point for deep venous drainage through the internal cerebral vein, yielding a Spetzler-Martin grade III.

**Figure 4:**
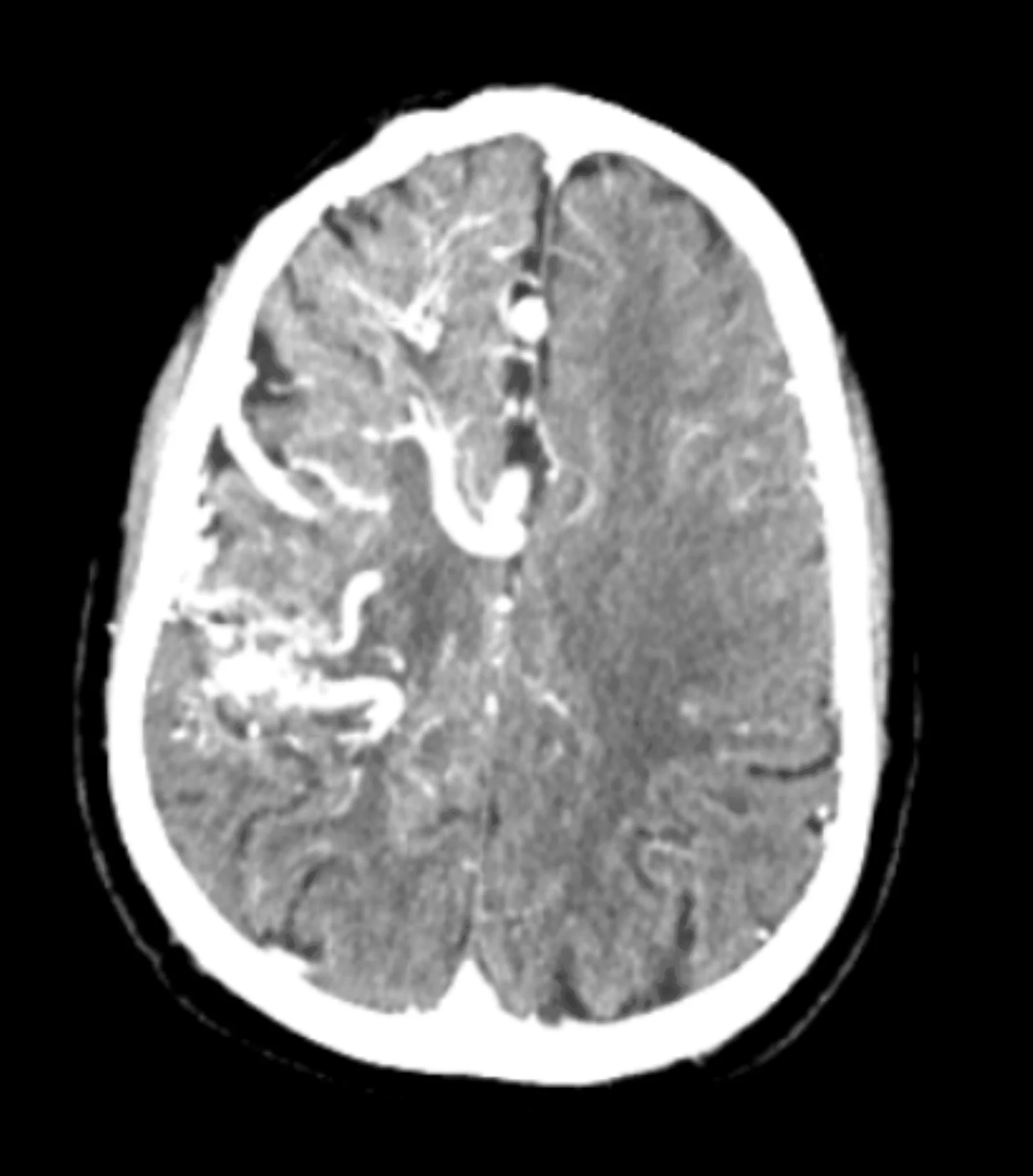
Follow-up contrast-enhanced CT scan, axial section, demonstrating stable appearance of the vascular malformations and the developmental venous anomaly (DVA), with no evidence of intracranial hemorrhage

The patient was subsequently lost to follow-up for two years. During this interval, the clinical course was marked by the onset of intermittent headaches, which prompted an emergency contrast-enhanced CT (Figure 4) to rule out hemorrhagic complications. Imaging showed no evidence of intracranial hemorrhage and demonstrated stability of all previously described vascular lesions, with no interval growth, new aneurysm formation, or new vascular abnormalities.

It should be noted that the right upper limb tremor is anatomically inconsistent with identified right hemispheric vascular lesions, and is therefore considered an incidental finding likely attributable to an independent neurological condition. The patient was referred for dedicated neurological workup; however, he was subsequently lost to follow-up, and no formal etiological diagnosis could be established.

Given the absence of hemorrhage, the stability of the lesions, the presence of a large DVA ensuring normal venous drainage, and the patient’s refusal of any invasive treatment, a conservative management strategy with clinical and radiological surveillance was recommended following multidisciplinary discussion between neuroradiology and neurosurgery teams.

## Discussion

Background

The coexistence of AVMs and DVAs represents an uncommon but clinically relevant vascular association. While DVAs are generally considered benign variants of venous drainage, their presence, in combination with AVMs, creates a complex and potentially unstable hemodynamic environment. In such cases, the DVA is no longer an incidental finding but a functionally critical component of the vascular network, with direct implications for both clinical behavior and management [1-3].

Epidemiology

DVAs are the most common intracranial vascular malformations, with a reported prevalence of 2-4%, whereas AVMs are relatively rare, with an incidence of approximately 1 per 100,000 population. The coexistence of these lesions remains uncommon and likely underrecognized. Nevertheless, available data suggest that combined AVM-DVA lesions may behave more aggressively than isolated AVMs, particularly with respect to hemorrhagic risk [2,3].

Pathophysiology

The pathophysiological relationship between AVMs and DVAs is primarily driven by hemodynamic interactions. AVMs generate high-flow arteriovenous shunting, increasing venous return and pressure within the draining system. DVAs, characterized by thin-walled veins and limited adaptive capacity, are particularly vulnerable to such changes, predisposing to venous hypertension, thrombosis, or rupture [1].

In the present case, the presence of two distinct right-sided AVMs (frontal and paracentral) sharing a hemispheric DVA likely resulted in a cumulative hemodynamic burden on the venous drainage system. This configuration is exceptionally rare and further complicates the vascular balance, as both lesions depend on a common venous outflow pathway. Such an arrangement may increase susceptibility to venous congestion and instability, even in the absence of overt hemorrhage.

Clinical presentation and hemorrhagic risk

Patients with AVM-DVA coexistence may present with hemorrhage, seizures, or focal neurological deficits. In our case, the initial presentation was atypical, consisting of right upper limb tremor associated with memory disturbances, without evidence of hemorrhage.

However, studies have reported a higher overall hemorrhagic prevalence in AVMs associated with DVAs (approximately 38%) compared with isolated AVMs (22.8%), whereas isolated DVAs are associated with a very low hemorrhagic risk, estimated at 0.15%-0.68% [1-3]. This increased risk is likely related to impaired venous drainage and elevated venous pressure within the DVA, particularly when it serves as a shared outflow pathway, as observed in our case.

Role of imaging

Imaging plays a central role in identifying and characterizing this vascular association. DVAs typically demonstrate a “caput medusae” appearance, with radially oriented medullary veins converging into a collector vein, best visualized during the venous phase [1]. In contrast, AVMs are defined by a nidus with early venous drainage and identifiable arterial feeders.

In our case, imaging was particularly valuable in demonstrating that both AVMs converged toward a common hemispheric venous drainage, highlighting the functional importance of the DVA. DSA remains the gold standard for detailed angioarchitectural assessment, allowing precise evaluation of the relationship between arterial inflow and venous outflow. MRI further contributes by assessing parenchymal integrity and excluding associated complications such as hemorrhage or venous infarction.

Management considerations

The coexistence of AVM and DVA significantly influences therapeutic decision-making. A fundamental principle is the strict preservation of the DVA, as it ensures venous drainage of normal brain parenchyma. Injury or occlusion of the DVA may result in catastrophic complications, including venous infarction, edema, or thrombosis [1,3].

As remarked in our review of the literature, the rare unfavorable outcomes were primarily related to treatment of both the AVM and the associated DVA, leading to venous hemorrhagic infarction and recurrent AVM bleeding. One of the earliest reports was published by Steiner and colleagues in 1993. They described a patient presenting with hemorrhage who underwent gamma knife radiosurgery for both lesions. The patient subsequently died 28 months after irradiation because of recurrent hemorrhage, most likely related to delayed occlusion of the DVA component [4].

In the present case, a conservative management strategy with close imaging surveillance was adopted. This decision was based on the absence of hemorrhage, the relatively mild clinical presentation, and the complex vascular configuration involving two AVMs sharing a hemispheric DVA. This configuration significantly increases the risk of venous complications in case of intervention, supporting a non-interventional approach.

When treatment is required, it should be directed exclusively at the AVM component using microsurgery, endovascular embolization, or radiosurgery, while preserving venous outflow integrity. Careful postoperative monitoring is also essential, as hemodynamic changes may predispose to DVA thrombosis.

Determining the optimal therapeutic strategy for arteriovenous malformations remains challenging, particularly regarding the management of unruptured lesions. The principal dilemma lies in balancing the potential benefits of preventive eradication against the risks associated with intervention. The randomized clinical trial ARUBA (A Randomised trial of Unruptured Brain Arteriovenous malformations) demonstrated that invasive treatment of unruptured AVMs was associated with a more than threefold increase in the risk of stroke or death compared with conservative management [5].

## Conclusions

The coexistence of multiple AVMs with a hemispheric DVA represents a rare and complex vascular configuration associated with significant hemodynamic implications and increased hemorrhagic risk. Even in the absence of hemorrhage, careful evaluation and long-term surveillance are essential. Preservation of the DVA remains the cornerstone of management to prevent potentially devastating venous complications. Given the extreme rarity and anatomical variability of combined AVM-DVA lesions, no single standardized management protocol can be established. Treatment decisions must be individualized, guided by multidisciplinary discussion, and tailored to the specific angioarchitecture, hemorrhagic risk profile, and clinical presentation of each patient.
